# An accessible and versatile deep learning-based sleep stage classifier

**DOI:** 10.3389/fninf.2023.1086634

**Published:** 2023-03-02

**Authors:** Jevri Hanna, Agnes Flöel

**Affiliations:** ^1^Greifswald University Hospital, Greifswald, Germany; ^2^German Center for Neurodegenerative Diseases, Standort Rostock/Greifswald, Greifswald, Germany

**Keywords:** sleep, deep learning, machine learning, classification, EEG

## Abstract

Manual sleep scoring for research purposes and for the diagnosis of sleep disorders is labor-intensive and often varies significantly between scorers, which has motivated many attempts to design automatic sleep stage classifiers. With the recent introduction of large, publicly available hand-scored polysomnographic data, and concomitant advances in machine learning methods to solve complex classification problems with supervised learning, the problem has received new attention, and a number of new classifiers that provide excellent accuracy. Most of these however have non-trivial barriers to use. We introduce the Greifswald Sleep Stage Classifier (GSSC), which is free, open source, and can be relatively easily installed and used on any moderately powered computer. In addition, the GSSC has been trained to perform well on a large variety of electrode set-ups, allowing high performance sleep staging with portable systems. The GSSC can also be readily integrated into brain-computer interfaces for real-time inference. These innovations were achieved while simultaneously reaching a level of accuracy equal to, or exceeding, recent state of the art classifiers and human experts, making the GSSC an excellent choice for researchers in need of reliable, automatic sleep staging.

## Introduction

Analysis of sleep stages for diagnosis of various sleep disorders, as well as analysis on more sophisticated microstructure of sleep like slow oscillations, spindles, and their coupling for research purposes (Rasch and Born, 2013 for review), has become an important goal in clinical and research context. Sleep consists of a rich diversity of neural and physiological stages, which typically unfold in semi-regular cycles throughout the sleep period. These stages have distinct signatures that can be measured with polysomnography (PSG), which includes the measure of neurophysiology with electroencephalogram (EEG), as well as ocular (EOG) and muscular (EMG) activity. Established guidelines (Rechtschaffen and Kales, 1973; Silber et al., 2007) allow for manual classification of PSG data into discrete sleep stages in 30 s increments, but this is a highly laborious process, requiring as much as 2 h to classify a single night’s sleep, even for a trained expert (Vallat and Walker, 2021). In addition to the costs of manual classification, there is also substantial disagreement between expert sleep stage scorers (70–80% agreement) and even within the same expert scorer at different times (90% agreement) (Rosenberg and Van Hout, 2013; Younes et al., 2016; Muto et al., 2018), which introduces a non-trivial degree of variability to both research findings and clinical diagnosis based on manual sleep staging. This variability is an inevitable consequence of the relative indeterminacy involved in applying sleep stage criteria to highly complex and variable human polysomnographic data. Sleep scorers make decisions on the basis of e.g., occipital alpha for a certain period of time, sleep spindles, K-complexes, certain types of eye movements, or overall EEG amplitude, to name just a few. On top of this, there are extensive contextual rules that specify under what circumstances one stage can follow another, adding a further layer of complexity and subjectivity. There is self-evidently wide interpretative discretion among sleep scorers as to how to identify these phenomena and how exactly to balance these rules against each other. The same indeterminacy also makes algorithmic sleep staging with traditional, analytical approaches difficult. Nevertheless, there have been many attempts, dating back to at least the 1990s, though these generally have not demonstrated robust generalizability [see Sun et al. (2017) for references and discussion].

Meanwhile however, in the previous decade, tens of thousands of hours of expert-scored PSGs have become publicly available (Zhang et al., 2018, sleepdata.org), and an explosion of algorithmic innovation and increased computing power has opened the possibility to train machine learning and deep learning models on these large datasets. Given the costliness and unreliability of manual scoring on the one hand, and the limitations of analytical approaches on the other hand, it is unsurprising that automatic sleep staging with machine/deep learning immediately became a field of intense focus and development. Deep learning in particular has proven to be very well-suited to solving many highly complex pattern recognition problems that had not been satisfactorily solved with prior methods (LeCun et al., 2015). In a short period of time, a large number of classifiers have been developed utilizing machine learning or deep learning to successfully score sleep stages [for review see Fiorillo et al. (2019)]. However, even though this has produced a significant improvement to the state of the art, there are still several drawbacks to most currently available sleep classifiers. As Vallat and Walker (2021) point out in the introduction of their own sleep classifier, YASA, these classifiers have barriers which make them less accessible, such as either (1) costing money or requiring expensive software to run (e.g., MATLAB), (2) requiring more technical knowledge to configure and use than many researchers have at their disposal, or (3) requiring data transmission to an external server. A further barrier to wide-spread adoption of automatic sleep stage classifiers for research purposes is the lack of transparency around the algorithms and code, or non-standardized reporting of classifier accuracy.

To remedy these shortcomings and provide high-quality, automatic sleep staging to a larger community of researchers, we introduce here the Greifswald Sleep Stage Classifier (GSSC), with the following overall goals in mind: First, as with YASA (Vallat and Walker, 2021), we have endeavored to produce a sleep stage classifier that is open source, freely available and not dependent on paid software, relatively easy to install and use, and can be run locally on any reasonably powered PC. Second, the GSSC was trained in order to achieve high performance also on less common electrode arrangements, including EOG only. Third, the GSSC has been designed such that it can be straightforwardly integrated into brain computer interfaces or closed loop brain stimulation systems with minimal processing overhead. Fourth, we sought to make improvements to the overall accuracy of the classifier in relation to the state of the art.

Usage of the GSSC is documented at: https://github.com/jshanna100/gssc/.

## Materials and methods

### Architecture

**FIGURE 1:**
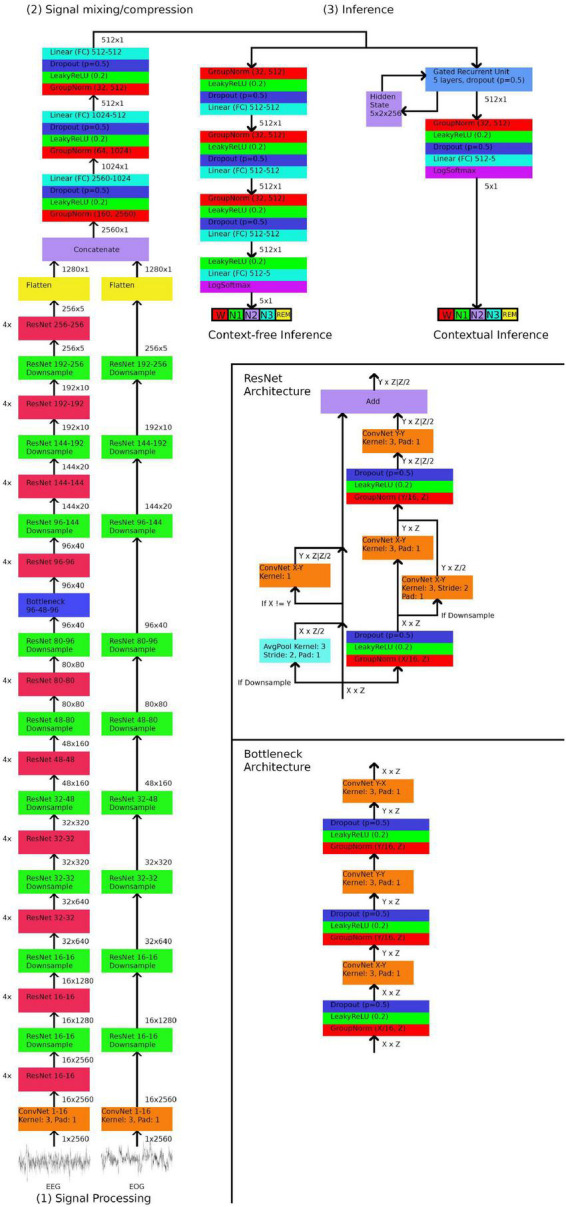
Neural network architecture. In stage (1), 30 s electroencephalogram (EEG)/ocular (EOG) signals downsampled to a length of 2,560 samples are input through a series of Resnets. The numbers in format X-Y indicate that the ResNet/ConvNet accepts X dimensional filters as input and outputs Y dimensional filters. In stage (2), the output of the ResNets are flattened into one-dimensional vectors, concatenated, and then mixed and compressed by three linear (fully-connected) layers into a vector of length of 512. In stage (3), the 512 length vector is passed onto one of two networks, depending on whether contextual or context-free inference is desired. Context-free inference consists of another three linear layers, and then a final layer which outputs a one-hot vector of length five, which encodes the five sleep stages. Contextual inference inputs the 512 length vector along with a hidden state into a Gated Recurrent Unit, which outputs a 512 length vector and an updated hidden state. A final linear layer outputs a one-hot vector of length five, which encodes the five sleep stages.

The GSSC uses a relatively simple architecture that requires minimal preprocessing or assumptions about relevant data features. The first part is a signal processing module that uses one-dimensional Resnets to convert the signal(s) into an abstract representation - expressed as a vector of size 1,280. Resnets are a form of convolutional neural network that utilize skipping connections to alleviate some of the typical problems encountered with deeper networks (He et al., 2016; Wu et al., 2019). Two separate Resnet stacks were trained; one for EEG and one for EOG. During prototyping, we found that the EOG Resnet does not benefit from significant depth, whereas the EEG Resnet improves significantly from added layers; here we added four Resnet blocks with each downsampling block. The next stage is a mixing and compression network, which–in the case of either just one EEG or just one EOG channel–compresses the vector of size 1,280 into a vector of size 512, and in the case of using both EEG and EOG channels, mixes and compresses the EEG and EOG vectors (2 × 1,280) into a vector of 512. Finally, this compressed vector of size 512 is either sent to a three-layer fully-connected network that decides the sleep stage, or passed onto a bidirectional Gated Recurrent Network (GRU) (Cho et al., 2014), which decides the sleep stage based on both the compressed vector and a hidden state which encodes the preceding/subsequent context. Network architecture is depicted schematically in Figure 1.

### Preprocessing

All PSGs were finite impulse response filtered with a bandpass of 0.3–30 Hz (one-pass, zero-phase, non-causal, filter length of 11.01 s, transition bandwidth of 0.3–7.5 Hz, 0.0194 passband ripple, 53 dB stopband attenuation). This band captures the most relevant oscillatory phenomena in human sleep, and safely excludes all line noise (50/60 Hz). Signals were Z-transformed on a per-channel, per-PSG basis. The right EOG channel was subtracted from the left EOG channels to form a single HEOG channel. All signals were downsampled to 85.33 Hz, which reduces a 30 s section to 2,560 samples, the length of signal input to the networks. Data were otherwise not cleaned or pruned in any way.

### Signal permutations and data augmentation

Every PSG in the training partition was trained multiple times, each time with a different signal permutation. Possible permutations include (1) an EEG channel, (2) the HEOG channel, (3) an EEG channel and HEOG channel together. Each possible EEG channel in a dataset was the basis of a permutation under conditions (1) and (3). In addition to these permutations, signals could also be flipped in polarity, i.e., by multiplying them by −1. This would mean for example that a dataset with two EEG channels and one EOG channel would have 14 possible permutations. The motivation for polarity flipping is to approximate the intrinsic relativity of EEG signals; every signal can easily change polarity simply by changing the EEG reference. Training under bipolarity, in addition to significantly augmenting the dataset size, has the goal of forcing the classifier to learn more abstract properties of the signal, resulting in a more flexible classifier that is likely to perform well under a larger variety of PSG recording set-ups and reference channels. Earlier prototypes also made use of the chin EMG channel, but it did not noticeably improve performance, similar to what was reported in Perslev et al. (2021). In the YASA classifier (Vallat and Walker, 2021), only one of the top 20 classification features was EMG based, ranking at 18th. This suggests that chin EMG contributes relatively little unique information to sleep stage classification.

### Training procedure

Neural networks were implemented within the PyTorch framework (v.1.10.2). For 20 training epochs, the 2,652 training PSGs were cycled through in random order. In order to fit within GPU memory constraints, PSGs were divided into 128 batches each with a roughly equal amount of contiguous, 30 s sections. A forward and backward pass was calculated on each batch, moving sequentially through the PSG. The forward and backward pass had two modes: context-free and context-aware. Both modes shared a common path for the first two stages, namely the signal processing and mixing/compression modules (see Architecture, Figure 1). After this point, they branched off, with the compressed vector going to the fully-connected, three-layer decision network in the case of context-free mode, which produced a one-hot vector of length 5, encoding for five possible sleep stages. This vector was compared against the correct sleep stage with a cross entropy loss function, and the loss was back-propagated through the decision network, mixing/compression, and signal processing modules. In the case of context-aware mode, the compressed vector and the previous hidden state was sent to the GRU network, which outputs a one-hot vector of length 5, again encoding for five possible sleep stages, and a new hidden state for the next batch. The loss was calculated in the same way as in context-free mode, and back-propagated through the GRU network, mixing/compression, and signal processing modules. After the losses had been back-propagated in both modes, the weights were updated, and the process was repeated for the next batch. For the first 30 s section of a PSG, the initial hidden state for the GRU network was set to all zeros. Because the models were trained in both modes simultaneously, the signal processing and mixing/compression modules learned to produce representations which could be used interchangeably with either the context-free decision network, or the context-aware GRU network. Weights were updated with the AdamW optimizer (Zhang, 2018) with hyperparameters beta1 = 0.9, beta2 = 0.999, and learning rate = 3e-5. Dropout (Hinton et al., 2012) was applied after every layer during training with a probability of 0.5. Because the frequency of sleep stages is severely imbalanced, it is necessary to provide the loss function with class weights to prevent the model from simply learning to guess blindly according to sleep stage probability. We adopted the weights used for the YASA algorithm (Vallat and Walker, 2021): Wake: 1, N1: 2.4, N2: 1, N3: 1.2, REM: 1.4, changing only the N1 weight slightly from 2.2 to 2.4 on the basis of prototyping.

### Data sets

We briefly list here the datasets and how they were implemented. For more information see sleepdata.org (Zhang et al., 2018). All datasets used the AASM system for sleep scoring (Silber et al., 2007), except the Sleep Health Heart Study (SHHS), which used Rechtschaffen and Kales (Rechtschaffen and Kales, 1973); the latter was rescored to be compatible with the other datasets. As we used only publicly available datasets, no ethical approval of our own was indicated; information on ethical approval of the individual data sets can be found in their corresponding articles, cited below.

### Sleep health heart study 2 (SHHS2)

The SHHS (Quan et al., 1997) is a large set of home PSGs assembled through 5 cohorts throughout the United States, primarily for the purpose of researching the connection between sleep-related breathing and cardiovascular disease. All participants were at least 40 years of age. We restricted ourselves here to the second phase of the project SHHS2, collected between 2001 and 2003, and used here a sample of 936 PSGs from the total 3,295 PSGs. Relevant electrodes include C3-A2, C4-A1, and left and right EOG. This study used the Rechtschaffen and Kales system, which we rescored to the AASM system (all N4 stages become N3).

### Cleveland family study (CFS)

The CFS (Redline et al., 1995) is a longitudinal (1990–2006) study focusing on sleep apnea in the United States. It features a particularly high proportion (46%) of Black American participants. We use here all 730 of the available PSGs. Relevant electrodes include C3-Fpz, C4-Fpz, and left and right EOG.

### Nationwide children’s hospital sleep databank (NCHSDB)

The NCHSDB (Lee et al., 2021) is a large pediatric dataset of PSGs collected in the United States, with most participants under the age of 10. We use here a sample of 665 of the 3,984 PSGs. Relevant electrodes include F3-M2, F4-M1, C3-M2, C4-M1, and left and right EOG.

### Wisconsin sleep cohort (WSC)

The WSC is a still ongoing longitudinal study focusing on various sleep disorders, collected in the United States. We use here a sample of 983 PSGs from the second stage of the project, collected with the Grass Comet Lab system (2009–present). Relevant electrodes include F3-M2, C3-M2, O1-M2, and left and right EOG. For more information see https://sleepdata.org/datasets/wsc and Young et al. (2009).

Information on datasets are also summarized in Table 1.

**TABLE 1:** Polysomnography datasets.

Dataset	Used/ Total	Age range	Pathologies
Sleep health heart study 2 (2001–2003)	936/3295	40+	Cardiovascular disease
Cleaveland family study	730/730	4–96	Sleep apnea
Wisconsin sleep cohort	983/8794	40–60	Primarily sleep apnea
Nationwide children’s hospital sleep databank	665/3984	Mostly < 15	Primarily sleep apnea or unspecified disorder

### Training, validation, and testing partitions

A combined total of 3,314 nights of manually scored PSGs, comprising 29,299 h and 3,515,889 individual 30 s epochs, derived from the four datasets listed above were used for training, validating, and testing the networks. Additionally, the DREEM dataset (Guillot et al., 2020) was used as a final test and point of direct comparison with a few of the most recent other classifiers. 80% (2,652 PSGs) of the full dataset were used for training. 10% (331) were used for validation (model prototyping, hyperparameter selection, assessment of overfitting), and a final 10% were used for testing; performance on these 10% as well as on the DREEM dataset are the basis for all reported results.

### Assessment measures

As a primary measure of performance, we use the Matthews Correlation Coefficient (MCC), which requires high true positives and negatives as well as low false positives and negatives to produce a good score, and is particularly well-suited for evaluating results on unbalanced datasets (Chicco and Jurman, 2020). In the interest of comparability with other studies as well as offering quick, intuitive results, we also report here F1 for each sleep stage, F1 macro, simple accuracy, Cohen’s Kappa, and confusion matrices [see Menghini et al. (2021) for discussion of these]. All of these metrics are calculated for each PSG separately. Medians across all PSGs rather than means are reported to prevent distortion from outlier PSGs.

### Permutation consensus

The classifier can infer sleep stage from any combination of EEG and EOG channels, or from only one of the two. It is therefore possible to make multiple inferences from the same PSG using different channel combinations. It also possible to calculate the certainty of that inference using the cross entropy of the log softmax vector of length 5 that is output by the classifier against the inferred sleep stage. Results indicated that inferences with high certainty also tended to be correct more often than lower certainty inferences (Supplementary Figure 1). By adopting the inference of the permutation with the highest certainty, we can increase the accuracy of the classifier.

### Selection of optimal network and testing

Throughout training, the performance of the classifier on training data continually improves. This does not necessarily indicate however that the final state of the network is the best one; overfitting on the training set can occur. To ascertain the optimal stopping point for training, we assessed classifier performance at the end of each training epoch on the testing dataset (331 PSGs), and identified the point at which performance peaked. This point was chosen as the optimal network which would be used in the final, testing step.

As a final confirmation of the performance of the model on unseen data, we assessed optimal network classifier performance on the testing dataset (331 PSGs). In addition to this, we also assessed performance on the DREEM dataset, both on healthy participants (*n* = 25) and those with sleep related breathing disorders (*n* = 55). The DREEM dataset was rated by five expert sleep scorers, and we assessed the classifier against the majority consensus of their scoring. In parallel to this, we assessed the YASA algorithm (Vallat and Walker, 2021) on both the testing dataset and the DREEM dataset for a direct comparison of the two. Finally, for the DREEM dataset only, we report the performance of two other state of the art classifiers, U-Sleep (Perslev et al., 2021) and that of Stephansen et al. (2018), using data kindly provided to us by Raphael Vallat (Vallat and Walker, 2021). In order to assess the flexibility of the GSSC and YASA classifiers, we assessed performance on all possible combinations of EEG/EOG/EMG channels. EMG combinations were implemented for YASA only, as GSSC does not make use of EMG channels, and combinations without EEG were implemented for GSSC only, as YASA requires the use of an EEG channel. Also, for the GSSC classifier we made use of the consensus of permutations assessment (see section Permutation consensus above).

## Results

### Training and model selection

During training, loss steadily declined and accuracy steadily increased; by the 20th training epoch these were near asymptote (Supplementary Figure 2). Performance on the validation set however oscillated up and down across the 20 training epochs, and did not reach convergence (see Supplementary Figure 3). For this reason, we selected three epochs that had both a high Matthews Correlation Coefficient and a high F1 Macro score. The reason for balancing across these two measures is that a high F1 Macro ensures that accuracy for all five sleep stages is relatively good; due to the severely unbalanced nature of sleep stages, a model could perform poorly in a less common sleep stage but still have a high overall MCC or accuracy. The weights from these three training epochs were averaged and the resulting weights were then used to infer sleep stages on the testing datasets. Weight averaging across multiple epochs throughout training has been shown to improve accuracy and generalization to unseen data (Izmailov et al., 2018).

### Context-aware and context-free inference

**FIGURE 2:**
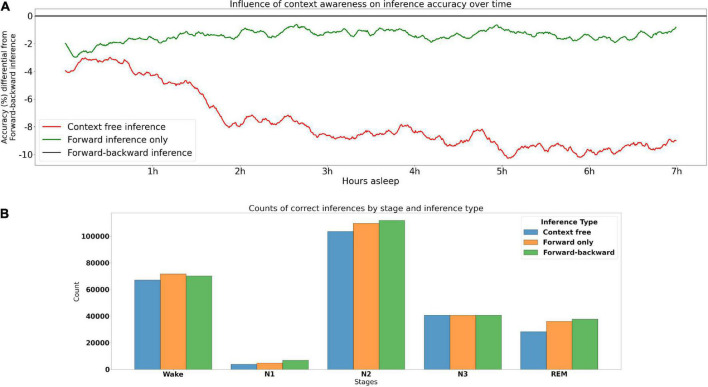
Contribution of contextual information to accuracy. **(A)** Accuracy of types of inference over time. The model performs optimally when making use of both preceding and subsequent epochs (forward-backward inference)–this optimal performance is the baseline at 0. The red and green lines indicate the loss in accuracy for context free inference and forward inference (preceding context only), respectively, across time for 355 PSGs in the testing set. **(B)** Accuracy of sleep stages under different types of inference, higher bars indicate more accurate.

To assess the influence that contextual information has on inference accuracy throughout the PSG, we calculated accuracy by PSG epoch across the 355 PSGs from the testing set (including DREEM) that had at least 875 30 s epochs (7.5 h). This was done under three contextual conditions: (1) the optimal, bidirectional inference–where for a given PSG epoch both the preceding and following context were taken into account–was used as the baseline. This type of inference would be used in offline PSG scoring. (2) In the forward inference, only the preceding context could be used for inference. This type of inference would be used in e.g., real-time/BCI inference. (3) Context free inference uses no context at all. Results are shown in Figure 2. These indicate that forward inference begins at around a 2% accuracy disadvantage relative to bidirectional inference, that linearly decreases to about 1% over the course of the PSG. Context free inference begins at a disadvantage somewhat below 4% relative to bidirectional inference, which sharply decreases to around an 8% disadvantage by around 2 h, then declines more slowly until around 5 h, after which the relative disadvantage seems to asymptote. These results underline the critical role of context in accurate sleep stage inference.

### Performance on DREEM dataset in relation to other classifiers

**FIGURE 3:**
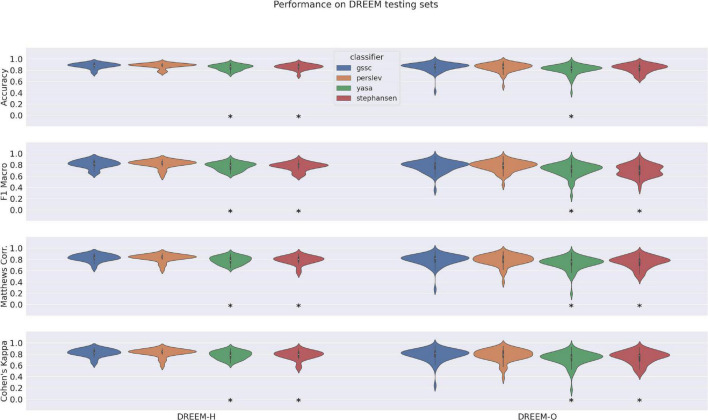
Violin plots of performance over DREEM dataset for four classifiers. Measures shown here include Accuracy, F1 Macro, Matthews Correlation Coefficient, and Cohen’s Kappa. Asterisks indicate significant differences from GSSC performance, as assessed by a linear mixed effects model with performance as the dependent variable, and classifier as a categorical factor.

Accuracy, F1 Macro, MCC, and Cohen’s Kappa scores for GSSC and three other recent classifiers are given in Figure 3. Supplementary Table 1 shows the exact numbers, and Supplementary Figure 4 shows the F1 scores for individual sleep stages. These indicate an accuracy advantage of 0.7% for Perslev et al. (2021) over GSSC on the Healthy cohort (*n* = 25), and an accuracy advantage of 2% for the GSSC over Perslev et al. (2021) on the Obstructive cohort (*n* = 55). These differences were tested statistically using a linear mixed effects model with performance as the dependent variable, and classifier as a categorical factor. This indicated no significant difference between the GSSC and Perslev et al. (2021), but significantly better performance than YASA and Stephansen et al. (2018). In summary, the GSSC performs at the current state of the art, and is at parity with Perslev et al. (2021), offering the highest possible accuracy.

**FIGURE 4:**
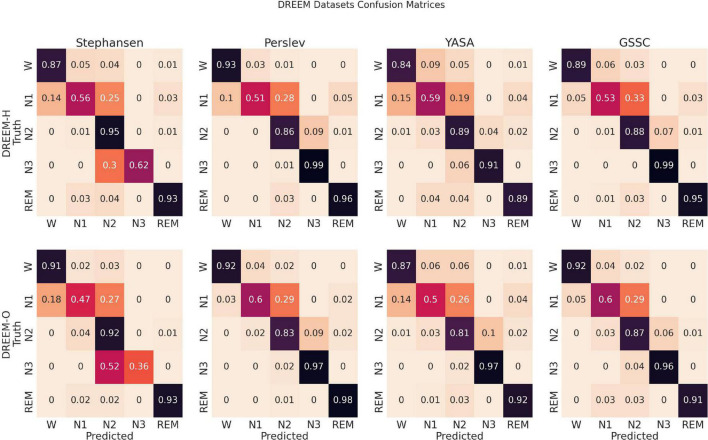
Confusion matrices. These row-normalized confusion matrices show the inferential behavior for four, recently developed, high performance classifiers, including the Greifswald Sleep Stage Classifier (GSSC). The diagonal indicates the accuracy, and off-diagonal elements show how the true sleep states tended to be misclassified. The first row shows results for the DREEM Healthy dataset (*n* = 25), and the second for the DREEM Obstructed dataset (*n* = 55).

Confusion matrices in Figure 4 show that, comparable to other classifiers, the main errors were confusing N1 for N2, and to a much lesser extent confusing N1 for Wake, N2 for N3, and vice versa. This pattern of confusion is consistent with that of both expert human raters and other automatic classifiers, and, aside from the expected, relatively poor performance on N1, shows high accuracy for all sleep stages.

### Discrepancy analysis

**FIGURE 5:**
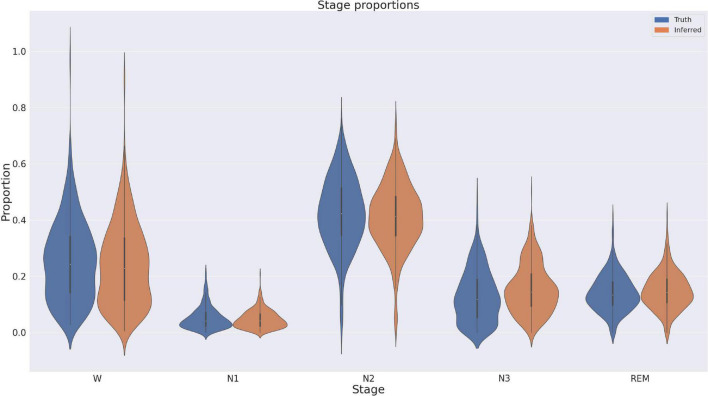
Proportions of stages in ground truth versus those inferred by the Greifswald Sleep Stage Classifier (GSSC). There is a slight bias of the GSSC toward N3.

We also directly compared the proportion of inferred sleep stages against the proportions of the expert consensus. These are depicted in Figure 5, and show generally very good correspondence, except for a small GSSC bias of a few percent toward N3. A linear mixed model with Proportion as the dependent variable and Stage and Expert/GSSC as factors confirmed this with an estimated interaction of N3 and Expert/GSSC, with a coefficient of 0.029 (2.9%) (*p* = 0.003). No other sleep stage proportion differed significantly between the GSSC and expert consensus. Finally, we counted sleep stage transitions for expert consensus and GSSC to ascertain whether the GSSC has a tendency to overly smooth or fragment the hypnogram. Expert consensus transitioned on average 0.125 (std. = 0.063) times per epoch, and the GSSC was slightly smoother at 0.119 (std. = 0.054). This difference was significant at *t* = 3.59, *p* < 0.001, with a small effect size (Cohen’s *d* = 0.099).

**FIGURE 6:**
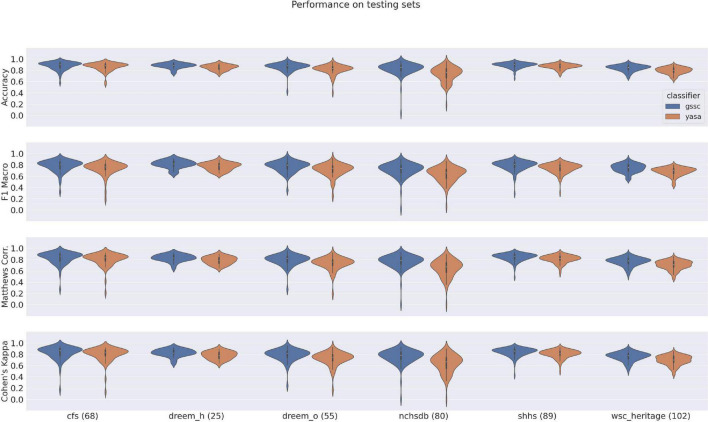
Violin plots of performance over all testing datasets for YASA and the Greifswald Sleep Stage Classifier (GSSC). Measures shown here include Accuracy, F1 Macro, Matthews Correlation Coeffecient and Cohen’s Kappa. Numbers in parentheses by the dataset name indicate the number of PSGs within that testing set.

**FIGURE 7:**
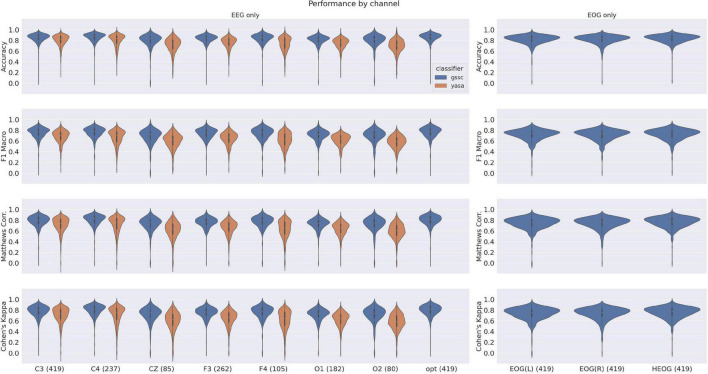
Violin plots for single-channel performance on the testing datasets for YASA and Greifswald Sleep Stage Classifier (GSSC). Measures shown here include Accuracy, F1 Macro, Matthews Correlation Coefficient and Cohen’s Kappa. Numbers in parentheses by the dataset name indicate the number of PSGs which had that channel available.

**FIGURE 8:**
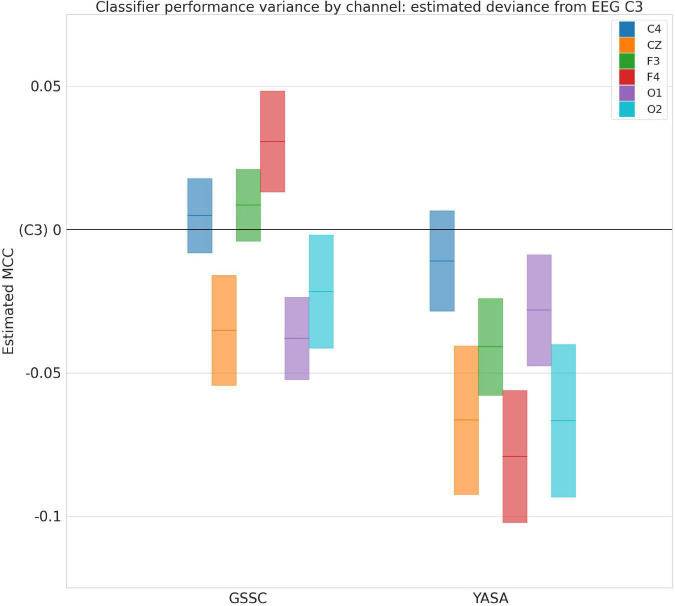
Performance variance across channels. Deviance in Matthews Correlation Coeffecient for different electroencephalogram (EEG) channels and Greifswald Sleep Stage Classifier (GSSC) and YASA classifiers from baseline of EEG C3, as estimated by a linear mixed effects model. Blocks show 95% confidence intervals around the estimated deviance (solid, colored lines).

The violin plots in Figure 6 show the performance across all testing sets for GSSC and YASA. Mean accuracy is well over 80% in all cases, and outperforms YASA by at least a few points across all datasets. Variance across PSGs also tends to be small except with CFS, NCHSDB, and DREEM-O; these three datasets were either composed of children or pathological populations. Figure 7 shows testing performance for different channels in isolation. All EEG channels perform above 80% mean accuracy. Left and right EOG as well as the difference of the two (HEOG) all have over 80% mean accuracy. Variance in performance across channels for GSSC and YASA was systematically compared with a liner mixed effects (LME) model that took accuracy for each individual EEG channel in each PSG in the testing set as data points, and estimated the fixed effects of channel and classifier on accuracy, with MCC performance on channel C3 as a baseline condition. Estimated effects with their confidence intervals are depicted in Figure 8. These show that for the GSSC, the performance of the different channels tended to cluster within a few percent above or below the baseline. For YASA on the other hand, only channel C4 performed comparably to their recommended C3 channel, and the others tended to be about 5% less accurate. This demonstrates the superior versatility of the GSSC classifier on diverse EEG channels.

### Light sleep

Many portable sleep tracking devices do not attempt to distinguish between N1 and N2 stages, but rather categorize these as Light sleep. An analysis with N1 and N2 concatenated into one stage can be found in the Supplementary Figures 5–8, and the numbers are found in Supplementary Table 2. Overall the GSSC was 91.5 and 91% accurate on the DREEM Healthy and Obstructed datasets, respectively. Perslev et al. (2021) was 91.5 and 89.5% accurate on the same datasets.

### Network interpretability

**FIGURE 9:**
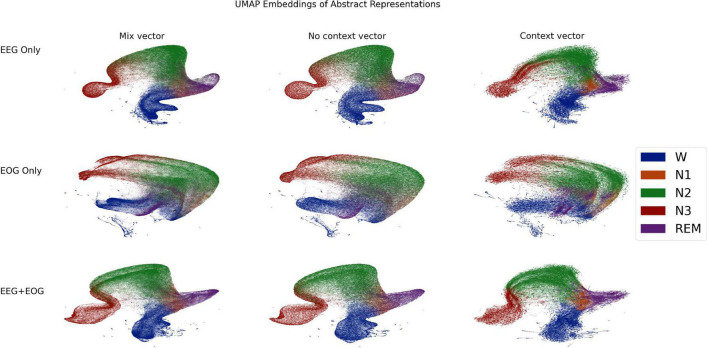
2 dimensional uniform manifold approximation and projection (UMAP) (Sainburg et al., 2021) embeddings of the vectors produced by the mixing/compression networks **(left column)**, the context-free inference **(middle column)**, and context-aware networks **(right column)** of the classifier. Each dot represents an embedding of a vector calculated from a single, 30 s epoch of a PSG from the testing set. Dots are color-coded by the sleep stage which was later inferred from that vector. See section Network Interpretability for details.

After the signal mixing/compression stage, the signal or signals have been transformed into a vector of length 512. This vector is an abstract representation of the signals which serves as the basis for the inference networks in the next stages: the context free inference network or the context sensitive GRU network. Even though these vectors are not the final outputs of the classifier, they nonetheless may contain interesting insights into the classifier’s internal properties. It is difficult to form intuitions about vectors in 512 dimensional space, but manifold learning algorithms can often embed high dimensional vectors into a much lower dimensional space while still preserving key elements of the high-dimensional topology. Here, we used Uniform Manifold Approximation and Projection (UMAP) (Sainburg et al., 2021) to transform the vectors for the entire testing set from 512 dimensional space to 2 dimensional space. In order to test the contributions of EEG and EOG, we did this separately for EEG only (C3), EOG only (Left EOG), and EEG and EOG together. Critical UMAP hyperparameters were nearest neighbors = 15, minimum distance = 0.1, and euclidean distance metric. In addition to the mix vectors, we also transformed vectors from the penultimate stage of both inference networks: the context-free network, and the GRU based context-aware network. These are also of length 512, and so can be readily compared with the mix vectors. Comparing the three vectors from different stages of processing within the classifier can give clues as to how these representations change. We used the Aligned UMAP technique to transform vectors from the three stages into a uniform space. The 2D embeddings are shown in Figure 9, color coded according to the sleep stage which was later inferred from them. Note that the manifold learning used only the vectors; sleep stage played no role in the manifold learning itself. These reveal the following insights: (1) Apart from N1, sleep stages have clearly demarcated regions. (2) With EOG however, the demarcations are visibly more degenerate than EEG. In particular the wake region bleeds diffusely into REM, N2, and N1 regions. (3) In EEG spaces, wake region transitions more sharply into N1 region, from which it may go on into REM or N2 regions. (4) In all spaces, N3 region may be entered only from N2 region. (5) In EOG only space, there is a cleft bisecting N2 and N3 regions, suggesting the classifier has two broad, classificatory schema within these stages. (6) There are a series of archipelagos along the “coast” of Wake region. These may be noisy epochs where the participant is moving around, which the classifier has learnt to associate with waking state. (7) The spaces for the no context vectors are practically identical to those of the mix vectors, indicating that the no-context inference network could in fact be mostly superfluous. (8) The N1 regions of the context vector spaces are more sharply segregated, especially in the EEG spaces, underlining again the critical role that context plays in identifying N1 as such.

## Discussion

We have developed an automatic sleep stage classifier that is free and requires no paid software, is easy to install and use, and can be run locally on a moderately well-powered PC. The YASA classifier (Vallat and Walker, 2021) also has these properties, but we have added features to our classifier that will make it preferable for many cases, including greater versatility, easy integration into brain computer interfaces (BCI), and overall improved accuracy. We discuss each of these in turn.

### Free availability and easy access

There has been rapid progress in the sophistication of automated sleep staging technologies, but it has not always been a priority to make this accessible. We developed GSSC within Python, which is free and can be run on any operating system, including Linux, which is also free, meaning that the GSSC can be run entirely on free software if desired. This offers high quality, automatic sleep staging to a much broader base of users who, either because of legal or resource constraints, are unable to use most of the other previously developed classifiers. Even researchers who do have the resources for paid software may nevertheless prefer to work within the free, open-source ecosystem for any number of reasons, not least of which because of the frictions that paid software often impose on their usage with e.g., licensing. The GSSC is at present built to work with MNE-Python (Gramfort et al., 2013, website:mne.tools), an M/EEG analysis Python package that is also free, open source, and community developed, though it could easily be adapted to work with any number of EEG analysis programs.

### Versatility

The training strategy we used has produced a versatile classifier that performs well with a broad array of possible electrode configurations. This allows the use of fewer EEG electrodes during recording, or to seamlessly move to backup channels if the more standard channels fail during a recording. GSSC is the only classifier of the four compared here to allow inference with only a single EOG channel, and furthermore with excellent accuracy (> 80%). This is concordant with the YASA classifier, which found that EOG absolute power was the single most important feature for sleep stage classification out of all the *a priori* defined features of the EEG/EOG/EMG signals that they used (Vallat and Walker, 2021). Highly accurate EOG-only inference is of particular use for more portable, 1–2 channel miniature systems designed for home testing (Gao et al., 2021).

### Brain-computer interfaces

Recent advances in the relevant hardware and software have fostered increased interest and development in brain-computer interfaces (BCI) (Abiri et al., 2019 for recent review). One special case of BCI that is of particular relevance here is the use of closed-loop stimulation, whereby some form of stimulation is given to the participant on the basis of their PSG/EEG activity; such systems have recently been successfully applied during sleep with the goal of modulating Slow Oscillations (e.g., Besedovsky et al., 2017; Fehér et al., 2021). For such systems targeting the enhancement of sleep oscillations, it is extremely useful to be able to assess the current sleep stage of the participant. GSSC is relatively easy to integrate into BCIs/closed-loop systems. This functionality is made possible in part by using a recurrent neural network for context awareness. Like human sleep stage scorers, all of the above cited classifiers make use of sleep stage context when performing inference. The GSSC implements context-awareness by use of a Gated Recurrent Network, which takes a hidden state as part of its input, and produces a new hidden state as part of its output. The hidden state contains the information that the classifier needs to make a context-informed decision on the present sleep stage. This makes it a natural fit for performing real time inference, as one can perform context-informed inference on each new 30 s epoch as it comes in, and does not need to observe the entire PSG at once.

### Accuracy

We compared the performance of the Greifswald Sleep Stage Classifier (GSSC) against three other recently developed, high-performance classifiers (Stephansen et al., 2018, U-Sleep, Perslev et al., 2021, YASA, Vallat and Walker, 2021) and found that it consistently outperformed Stephansen et al. (2018) and YASA (Vallat and Walker, 2021), and was at parity with U-Sleep (Perslev et al., 2021). This places GSSC at the current state of the art.

### Limitations

One disadvantage that GSSC has compared to YASA is speed. YASA can infer an entire night’s PSG in a few seconds, while GSSC requires somewhat more time, even with a GPU. With a CPU, inference time can go into the minutes for a full night–however this time can be significantly reduced by opting for a specific channel constellation rather than using the permutation consensus, likely at the cost of 1–2 percent accuracy. Researchers who have limited time and/or computing power, use conventional PSG setups and electrodes, and do not need the highest level of available accuracy might consider using YASA. Otherwise GSSC would be preferable for the reasons described above.

### Outlook

There are a number of things which should be improved in future versions of the GSSC. First, performance on the validation dataset over 20 training epochs did not converge, but rather oscillated continually (see Supplementary Figure 3). An average of the best three epochs produced excellent results on the testing sets, but it would nevertheless be preferable for the classifier to converge on a stable solution. Second, the classifier was trained on only 2,652 PSGs from four datasets. This is somewhat less than YASA, a non-deep learning classifier (3,163 PSGs, seven datasets), and a small fraction of what was used for the deep learning-based U-Sleep (19,924 PSGs, 21 datasets). Training on more data may solve the non-convergence issue mentioned above, and also yield a non-trivial accuracy increase; on the other hand, performance at the current state of the art may already be near the intrinsic limits of how accurate sleep staging can be, given the relative indeterminacy of sleep staging criteria, and that machine/deep learning classifiers are trained on manually scored data, which are themselves quite variable (Rosenberg and Van Hout, 2013; Younes et al., 2016; Muto et al., 2018). Third, during prototyping, we found good performance on four-layer Resnets for the EEG channel, and one-layer Resnets for the EOG. This is unsurprising given how much more complex a brain signal is from an ocular muscle signal, but there may be space to more thoroughly fine-tune these layer numbers. Finally, the weights used to adjust the loss function for the severe imbalance of sleep stages could potentially be improved. We have simply adopted the ones reported for YASA, with a minimal change to the N1 weight (Vallat and Walker, 2021), and they have yielded excellent results, but some small adjustments could prove beneficial, particularly with regard to the small N3 bias.

Finally, the results of the interpretability analysis have also yielded some interesting insights into the internal representations of the classifier. Future work can explore for example how these insights might allow for improved network architectures, or to provide more detailed inferential information, i.e., why a given epoch may have been inferred as such.

## Conclusion

The Greifswald Sleep Stage Classifier (GSSC) is free, open source, easy to install and use, offers state of the art accuracy, and performs well for all reasonable channel combinations, including only a single EOG channel. It is particularly well-suited to real time inference (BCI, closed-loop stimulation). These features render the GSSC an excellent candidate for becoming a standard tool for polysomnographers.

## Data availability statement

The original contributions presented in this study are included in this article/Supplementary material, further inquiries can be directed to the corresponding author.

## Author contributions

JH conceived, designed, tested the classifier, and wrote the manuscript. AF provided the supervision, funding, and wrote the manuscript. Both authors contributed to the article and approved the submitted version.
