# Supplementary material for: An accessible and versatile deep learning-based sleep stage classifier
Source: Front Neuroinform. 2023 Mar 2;17:1086634. doi: 10.3389/fninf.2023.1086634 (PMC10017438; doi:10.3389/fninf.2023.1086634)
Supplement: Supplementary file 1 [file Data_Sheet_1.pdf]

Supplementary Information

	GSSC H	GSSC O	YASA H	YASA O	Perslev H	Perslev O	Stephansen H	Stephansen O
Acc	88.8 +/- 4.8	88.1 +/- 5.8	86.2 +/- 7.3	83.2 +/- 6.0	89.5 +/- 2.2	86.1 +/- 8.1	85.9 +/- 5.8	84.9 +/- 9.3
MCC	82.5 +/- 6.8	81.8 +/- 10.3	78.0 +/- 10.2	75.6 +/- 9.9	84.3 +/- 3.5	79.4 +/- 11.1	80.1 +/- 8.3	77.5 +/- 11.7
CK	82.1 +/- 6.9	81.4 +/- 10.9	77.8 +/- 10.5	74.5 +/- 10.9	84.2 +/- 3.5	78.5 +/- 11.8	79.5 +/- 9.7	77.2 +/- 12.6
F1 Macro	81.8 +/- 7.9	79.9 +/- 10.6	78.7 +/- 11.1	74.0 +/- 11.6	83.3 +/- 4.5	79.4 +/- 10.4	78.2 +/- 5.5	69.8 +/- 15.9
F1 Wake	90.2 +/- 7.7	92.9 +/- 7.1	83.0 +/- 15.1	85.5 +/- 13.1	91.9 +/- 8.3	92.7 +/- 5.8	86.7 +/- 9.9	88.3 +/- 7.9
F1 N1	56.2 +/- 21.1	45.7 +/- 22.7	50.0 +/- 12.3	40.5 +/- 18.9	60.6 +/- 13.1	53.3 +/- 16.7	51.8 +/- 20.2	41.3 +/- 18.5
F1 N2	90.9 +/- 5.6	89.7 +/- 7.1	88.4 +/- 5.0	86.1 +/- 5.9	89.7 +/- 5.3	87.6 +/- 8.4	89.4 +/- 5.6	88.4 +/- 7.8
F1 N3	88.1 +/- 11.9	81.4 +/- 29.7	88.6 +/- 13.7	75.9 +/- 23.5	85.2 +/- 13.8	76.4 +/- 33.8	75.3 +/- 23.5	67.3 +/- 69.6
F1 REM	94.3 +/- 4.6	93.3 +/- 5.1	92.7 +/- 10.2	89.2 +/- 10.5	94.1 +/- 5.0	93.6 +/- 6.5	91.5 +/- 5.8	90.3 +/- 15.7

Table S1: Performance of the GSSC, YASA, U-Sleep (Perslev et al. 2021) and Stephansen et al. (2018) on the DREEM Health and Obstructive datasets. Measures include Accuracy (Acc), Matthews Correlation Coefficient (MCC), Cohen's Kappa (CK), F1 Macro and F1 scores for each individual stage. These are depicted graphically in Figs. 3 and S4.

	GSSC H	GSSC O	YASA H	YASA O	Perslev H	Perslev O	Stephansen H	Stephansen O
Acc	91.5 +/- 4.0	91.0 +/- 5.8	89.3 +/- 7.1	87.3 +/- 6.6	91.5 +/- 3.1	89.5 +/- 8.0	88.7 +/- 5.8	88.7 +/- 8.5
MCC	86.5 +/- 8.2	84.3 +/- 9.9	82.1 +/- 12.4	79.2 +/- 9.6	86.2 +/- 5.4	83.7 +/- 12.0	83.0 +/- 8.6	80.7 +/- 11.9
CK	86.2 +/- 8.2	84.1 +/- 10.0	81.4 +/- 12.5	78.9 +/- 10.6	86.1 +/- 5.2	83.4 +/- 13.8	82.3 +/- 10.4	80.6 +/- 13.5
F1 Macro	88.5 +/- 6.4	88.7 +/- 12.3	86.7 +/- 10.6	82.8 +/- 12.8	89.5 +/- 3.7	86.5 +/- 11.4	85.3 +/- 10.1	77.1 +/- 18.5
F1 Wake	90.2 +/- 7.7	92.9 +/- 7.1	83.0 +/- 15.1	85.5 +/- 13.1	91.9 +/- 8.3	92.7 +/- 5.8	86.7 +/- 9.9	88.3 +/- 7.9
F1 Light	92.4 +/- 4.7	91.8 +/- 6.1	89.0 +/- 5.5	87.9 +/- 5.8	91.5 +/- 3.1	89.9 +/- 6.8	89.9 +/- 5.1	89.8 +/- 6.8
F1 N3	88.1 +/- 11.9	81.4 +/- 29.7	88.6 +/- 13.7	75.9 +/- 23.5	85.2 +/- 13.8	76.4 +/- 33.8	75.3 +/- 23.5	67.3 +/- 69.6
F1 REM	94.3 +/- 4.6	93.3 +/- 5.1	92.7 +/- 10.2	89.2 +/- 10.5	94.1 +/- 5.0	93.6 +/- 6.5	91.5 +/- 5.8	90.3 +/- 15.7

Table S2: Performance of the GSSC, YASA, U-Sleep (Perslev et al. 2021) and Stephansen et al. (2018) on the DREEM Health and Obstructive datasets, with N1 and N2 collapsed into a single, "Light" stage. Measures include Accuracy (Acc), Matthews Correlation Coefficient (MCC), Cohen's Kappa (CK), F1 Macro and F1 scores for each individual stage. These are depicted graphically in Figs. S7 and S8.

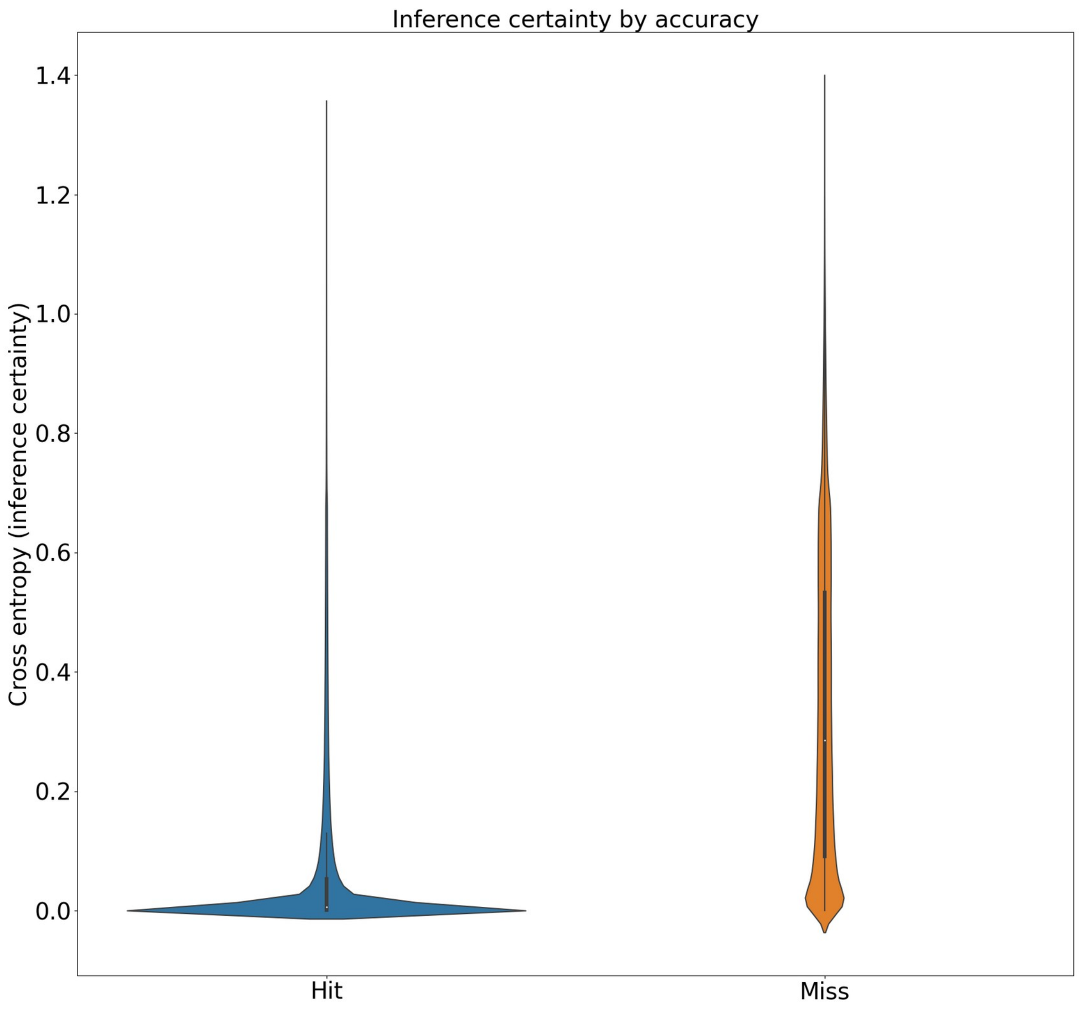

Figure S1: Relationship of inference certainty and accuracy. Violin plots show cross entropy loss of inferred sleep stage against the log softmax output of the classifier, which can be understood as an inverse index of the classifier's certainty about its inference. Accurate inferences ("Hit") tend to have much more certainty than inaccurate inferences ("Miss").

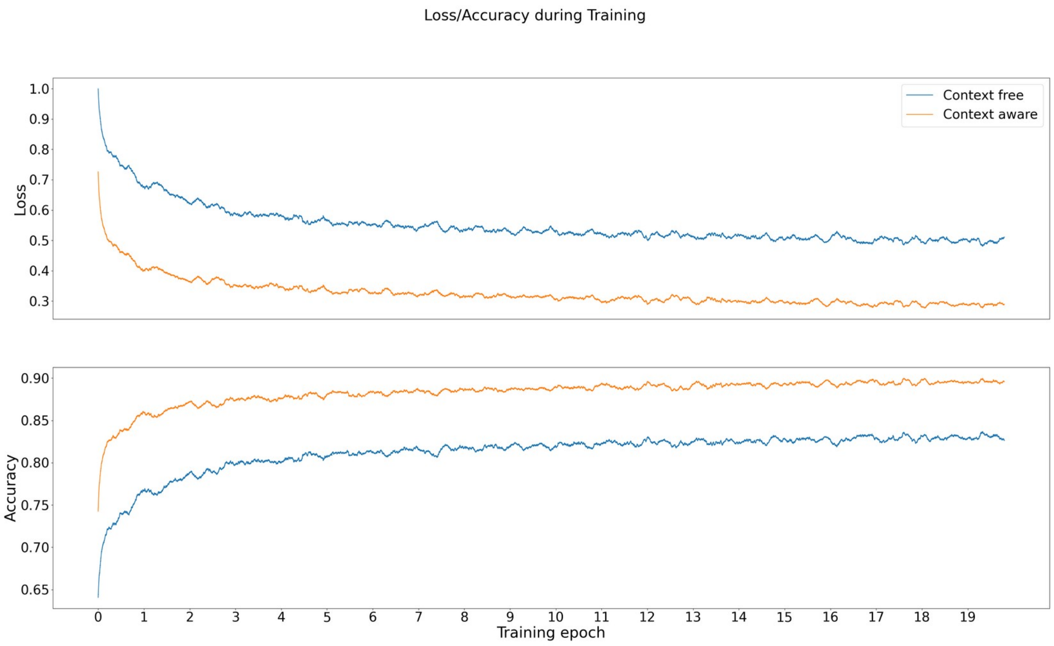

Figure S2: Loss and accuracy across 20 training epochs.

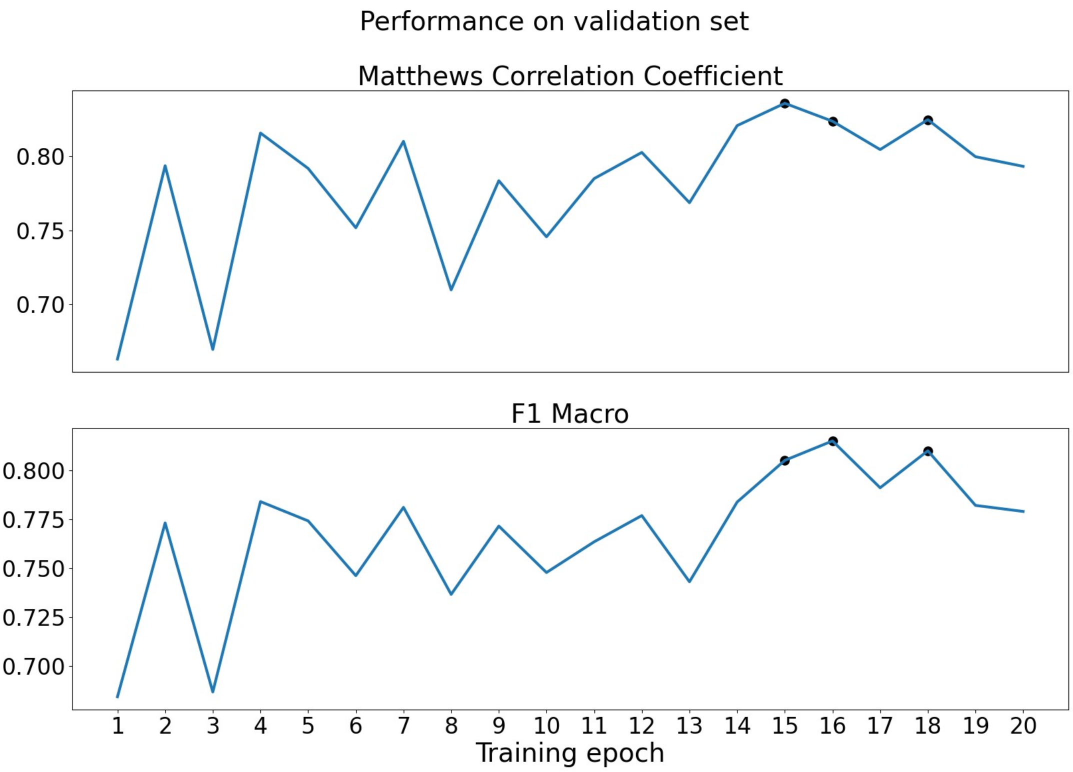

Figure S3: Performance on the validation set as assessed by MCC and F1 Macro. The MCC is a good assessment of overall performance, while the F1 Macro requires good performance on all possible classes. Because the model did not converge on the test set, three points were selected that had high scores on both measures, and the network weights for these three points were averaged. This network was then applied to the validation set to produce the results reported in this paper.

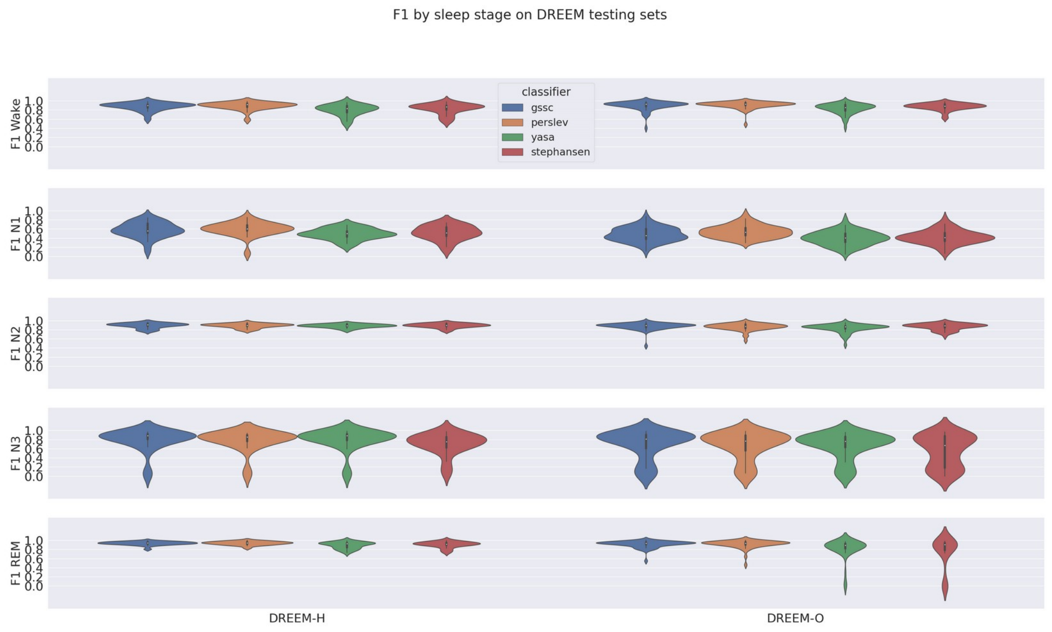

Figure S4: F1 measures for four classifiers for the five possible sleep stages on the DREEM testing datasets.

N1+N2=Light Analysis

These figures depict classifier(s) performance with N1 and N2 consolidated into a single sleep stage (Light).

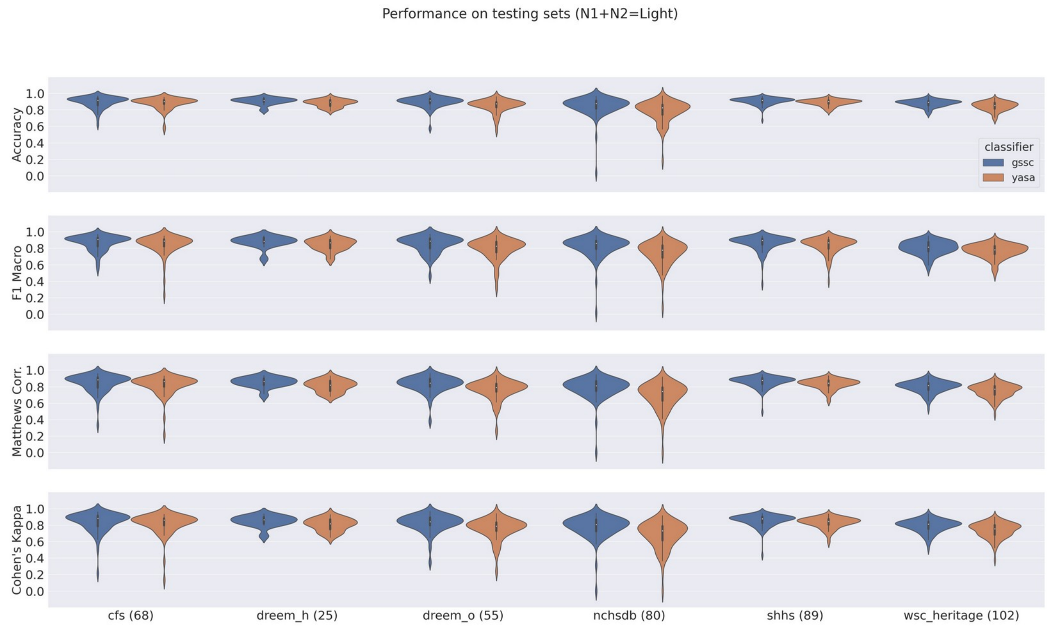

Figure S5: Violin plots of performance over DREEM dataset for four classifiers. Measures shown here include Accuracy, F1 Macro, and Matthews Correlation Coeffecient. Asterisks indicate significant differences from GSSC performance, as assessed by a linear mixed effects model with performance as the dependent variable, and classifier as a categorical factor. Numbers in parentheses by the dataset name indicate the number of PSGs within that testing set.

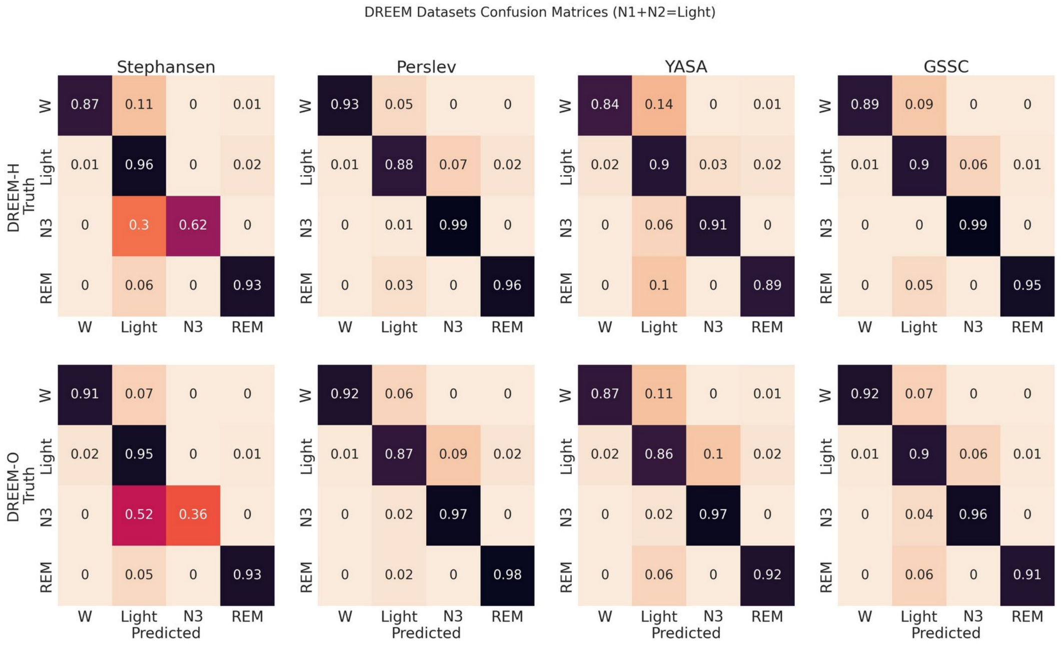

Figure S6: Confusion matrices. These row-normalised confusion matrices show the inferential behaviour for four, recently developed, high performance classifiers, including GSSC. The diagonal indicates the accuracy, and off-diagonal elements show how the true sleep states tended to be misclassified. The first row shows results for the DREEM Healthy dataset (n=25), and the second for the DREEM Obstructed dataset (n=55).

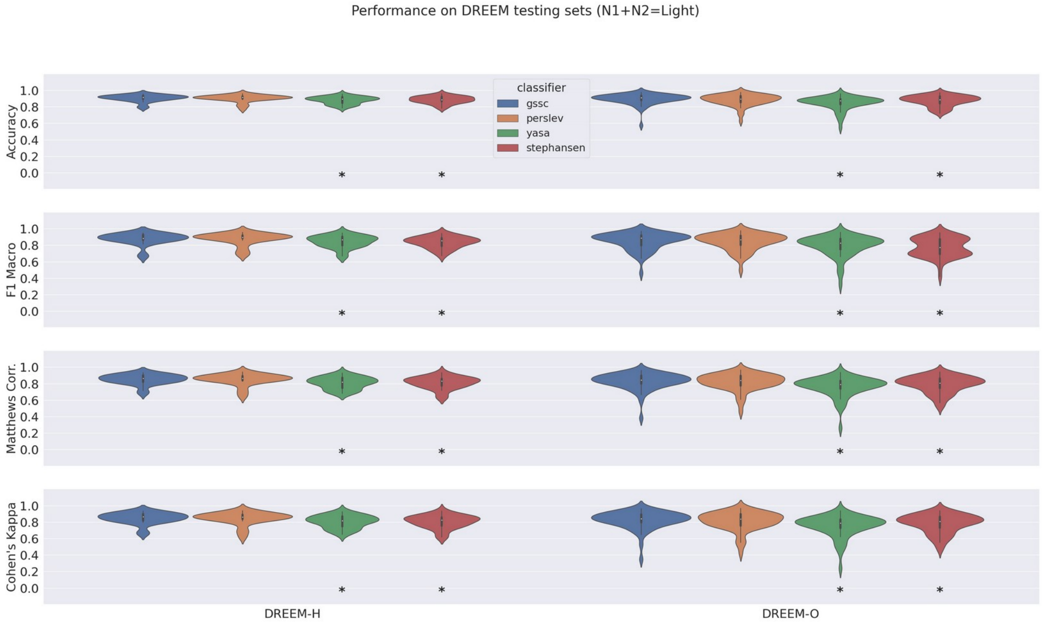

Figure S7: Violin plots of performance over all testing datasets for YASA and GSSC. Measures shown here include Accuracy, F1 Macro, and Matthews Correlation Coeffecient and Cohen's Kappa.

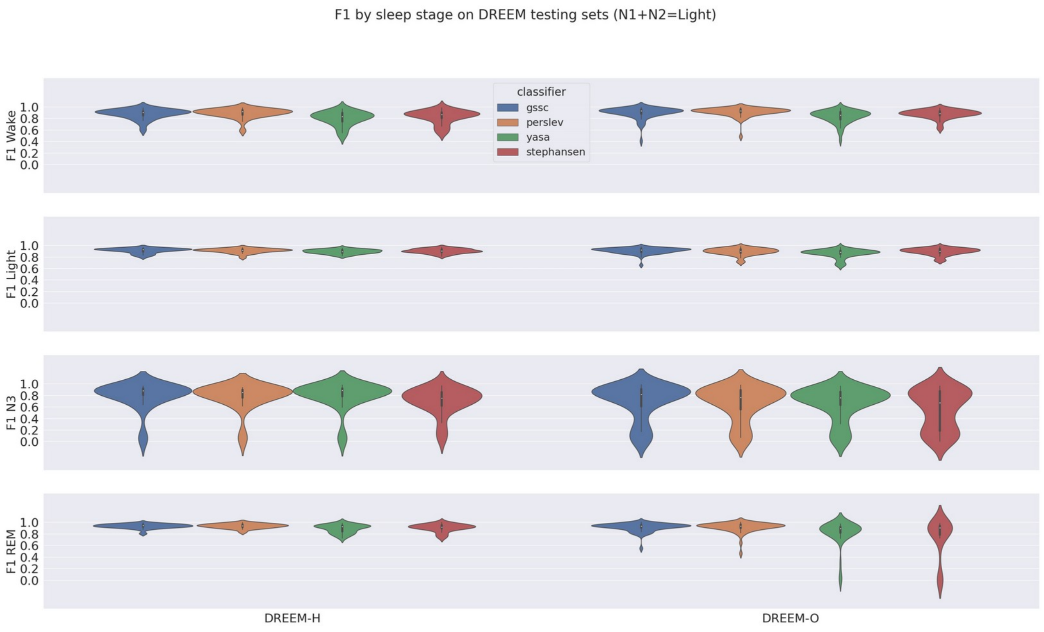

Figure S8: F1 measures for four classifiers for the NREM sleep stages on the DREEM testing datasets.
